# Dysregulation in IFN-γ signaling and response: the barricade to tumor immunotherapy

**DOI:** 10.3389/fimmu.2023.1190333

**Published:** 2023-05-18

**Authors:** Jiashu Han, Mengwei Wu, Ziwen Liu

**Affiliations:** ^1^ Chinese Academy of Medical Sciences and Peking Union Medical College, Beijing, China; ^2^ Department of General Surgery, Peking Union Medical College Hospital (CAMS), Beijing, China

**Keywords:** interferon, immunotherapy, immunoresistance, ISG (interferon stimulated genes), IFNGR, JAK - STAT signaling pathway, IFN-γ

## Abstract

Interferon-gamma (IFN-γ) has been identified as a crucial factor in determining the responsiveness to immunotherapy. Produced primarily by natural killer (NK) and T cells, IFN-γ promotes activation, maturation, proliferation, cytokine expression, and effector function in immune cells, while simultaneously inducing antigen presentation, growth arrest, and apoptosis in tumor cells. However, tumor cells can hijack the IFN-γ signaling pathway to mount IFN-γ resistance: rather than increasing antigenicity and succumbing to death, tumor cells acquire stemness characteristics and express immunosuppressive molecules to defend against antitumor immunity. In this review, we summarize the potential mechanisms of IFN-γ resistance occurring at two critical stages: disrupted signal transduction along the IFNG/IFNGR/JAK/STAT pathway, or preferential expression of specific interferon-stimulated genes (ISGs). Elucidating the molecular mechanisms through which tumor cells develop IFN-γ resistance help identify promising therapeutic targets to improve immunotherapy, with broad application value in conjugation with targeted, antibody or cellular therapies.

## Introduction

To date, over twenty distinct interferon (IFN) genes and proteins have been identified, typically categorized into three classes: Type I IFN (IFN-α and IFN-β), Type II IFN (IFN-γ), and Type III IFN (IFN-λ). IFN-γ is a notably different member characterized by unique receptor activity and distinct intracellular signaling pathway: type I IFNs depends on the interferon-stimulated gene factor-3 (ISGF3) complex containing STAT1/STAT2/IRF9, which binds to interferon-sensitive response elements (ISREs), whereas the phosphorylated signal transducer and activator of transcription 1 (STAT1) homodimer downstream of IFN-γ binds to interferon-gamma activation sites (GASs). However, these members share common interferon-stimulated genes (ISGs), but also have distinct gene profiles (1). For example, IFN-γ preferentially induces the expression of IRF1, while HIF-1 responses primarily to IFN-β (2). IFN-γ signaling and ISG expression is marked by extensive interaction with regulatory molecules, epigenetic modifications and chromatin remodeling, constituting multiple positive and negative feedbacks (3, 4). IFN-γ also produce variable effects at different duration and concentration of exposure, with acute exposure to high concentrations of IFN-γ leading to growth arrest and apoptosis, while chronic exposure to low concentrations promotes cell survival (5). The IFN-γ signaling pathway has extensive crosstalks with the PI3K, MAPK/p38, and other cellular pathways (6). Collectively, these mechanisms maintain immunological homeostasis, ensuring an effective immune response while keeping the immune activity in check to avoid damage from over activation. However, these regulatory mechanisms can also be exploited by tumors to mount IFN-γ resistance.

## IFN-γ in tumor

### Source

IFN-γ in tumors is primarily produced by activated immune cells, particularly NK, cytotoxic CD8+ and Th1 cells (7, 8). Evidence also suggests that macrophages, dendritic cells (DC), and innate B cells can produce IFN-γ (9–13). While tumor cells have been reported to rely on autocrine secretion of IFN-β (5, 14), further investigation is required to determine whether tumor cells secrete IFN-γ (15). IFN-γ production is stimulated by specific cytokines, including interleukin-12 (IL-12), interleukin-18 (IL-18), and macrophage colony-stimulating factor (M-CSF) (9, 16, 17), as well as receptor signaling by activating natural killer cell receptors (NKRs) and CD16 in NK cells and the T cell receptor (TCR) in T cells (17). These stimulations activate the Src/MAPK/ERK/p38 pathway (18), ultimately inducing IFNG expression through transcription factors STAT4, T-bet, AP-1, Eomes, Fos, and Jun (18, 19). Notably, GAS is present in the promoter IRF1 (20, 21), STAT1, IFNGR, and IFN-γ (22), imposing positive feedback and self-sustained inflammation (3, 19). The most recent report identified the interaction between GATA3 and CNS-28 silencer as a restrainer of IFN-y mRNA transcription by diminishing enhancer-promoter interactions within the gene locus (23).

### Effect

IFN-γ is known to regulate the expression of hundreds of ISGs, whose differential expression patterns confer various effects including growth suppression, induction of apoptosis, but also activation and secretion of pro-inflammatory cytokines, and other functions such as expression of immunosuppressive genes. However, the mechanisms underlying these opposing effects under the same set of signals, receptors, and main signaling molecules require further investigation.

#### Immune cells

In CD8 T cells, IFN-γ is crucial in T cell expansion (24), induces the differentiation of precursor T cells into effector T cells (25), further enhances their cytotoxicity and motility (26), and participates in the formation of immunological memory (27). In CD4 T cells, IFN-γ is pro-Th1 and antitumor, repressing Th2 and Th17 polarization (7, 28). In NK cells, IFN-γ induces CXCR3 expression to allow tumor infiltration (29) and TRAIL expression to promote cytotoxicity (30). In myeloid cells, IFN-γ promotes polarization toward the inflammatory cDC1 (31) and M1 (7, 32, 33). In B cells, IFN-γ not only cooperates with IL-12 to mediate class switching (34), but also aids germinal center formation through BCL6 (35).

Interestingly, IFN-γ also exerts suppressive effects on certain immune cells, producing both wanted and unwanted effects. IFN-γ stimulation generates ‘fragile’ regulatory T cells (Tregs) with impaired suppressive activity yet maintained Treg phenotype in terms of FOXP3+ (7, 36). However, chronic exposure and constitutive signaling of IFN-γ impair T cell functions and lead to exhaustion (37), not only through the well-studied induction of IDO1 and PD-L1 expression, but also through direct inhibition of T cell stemness, proliferation, clonal diversity, and maintenance (38–40), along with pro-apoptotic effect in the contraction phase through FAS and BIM (41, 42). Additionally, IFN-γ inhibits memory formation by limiting IL-7Ra in models of influenza virus infection (42).

#### Tumor cells

In general, IFN-γ exerts antitumor actions in two ways: directly through growth inhibition and indirectly through increased killing by immune cells (43). IFN-γ has growth-inhibitory effect through cell death induction by apoptosis (44–46) and necroptosis (47), and growth arrest by senescence (48–51). The immunostimulatory effects of increased MHC-I, antigen presentation, and antigenicity has been extensively characterized in both infection and tumor models (52).

On the other hand, IFN-γ itself and ISG signatures are pro-tumor in various cancer types including breast cancer (46, 53) and glioblastoma (54). Mechanistically, IFN-γ directly acts on tumor cells to maintain survival (BCL2 and surviving expression) and stemness (55–57), decreases antigen presentation (58), enhances the expression of immunosuppressive molecules markedly ICBs and IDO (58–60), and leads to infiltration of tumor-associated neutrophil (46). Additionally, chronic exposure to IFN-γ inevitably induce immunosuppressive cells, such as myeloid-derived suppressor cells (MDSCs), Tregs and exhausted T cells, ultimately leading to immune evasion and tumor growth.

### Resistance

Ideally, IFN-γ stimulation would force the tumor cells to increase their antigenicity and secrete pro-inflammatory cytokines, along with growth arrest and pro-apoptosis. However, tumor cells can suppress these immunogenic ISGs and favor the expression immunosuppressive and pro-tumor ISGs instead. IFN-γ resistance is a major obstacle in cancer immunotherapy, offering protection against T cell cytotoxicity and ICBs, and allowing tumor cells to exploit IFN-γ signaling for survival advantage rather than succumb to antitumor immunity. Elucidating the mechanisms of interferon resistance and developing new strategies to tackle it are important areas of research, with great potential to identify effective therapeutic target as adjuvant to current cancer immunotherapy. Briefly, there are two principle mechanisms though which tumor cells mount IFN-γ resistance: 1) disruption in the primary signaling pathway, IFNGR/JAK/STAT, responsible for effector functions of IFN-γ stimulation; and 2) epigenetic or regulatory background leading to favorable expression of some ISGs over others. Therefore, we next summarize recent findings regarding the molecular mechanisms behind the aberrant IFN-γ response in tumor cells. It is important note the limitation in generalizability across different experimental models, as these mechanisms are often context-specific.

## IFN-γ signaling

**Figure 1:**
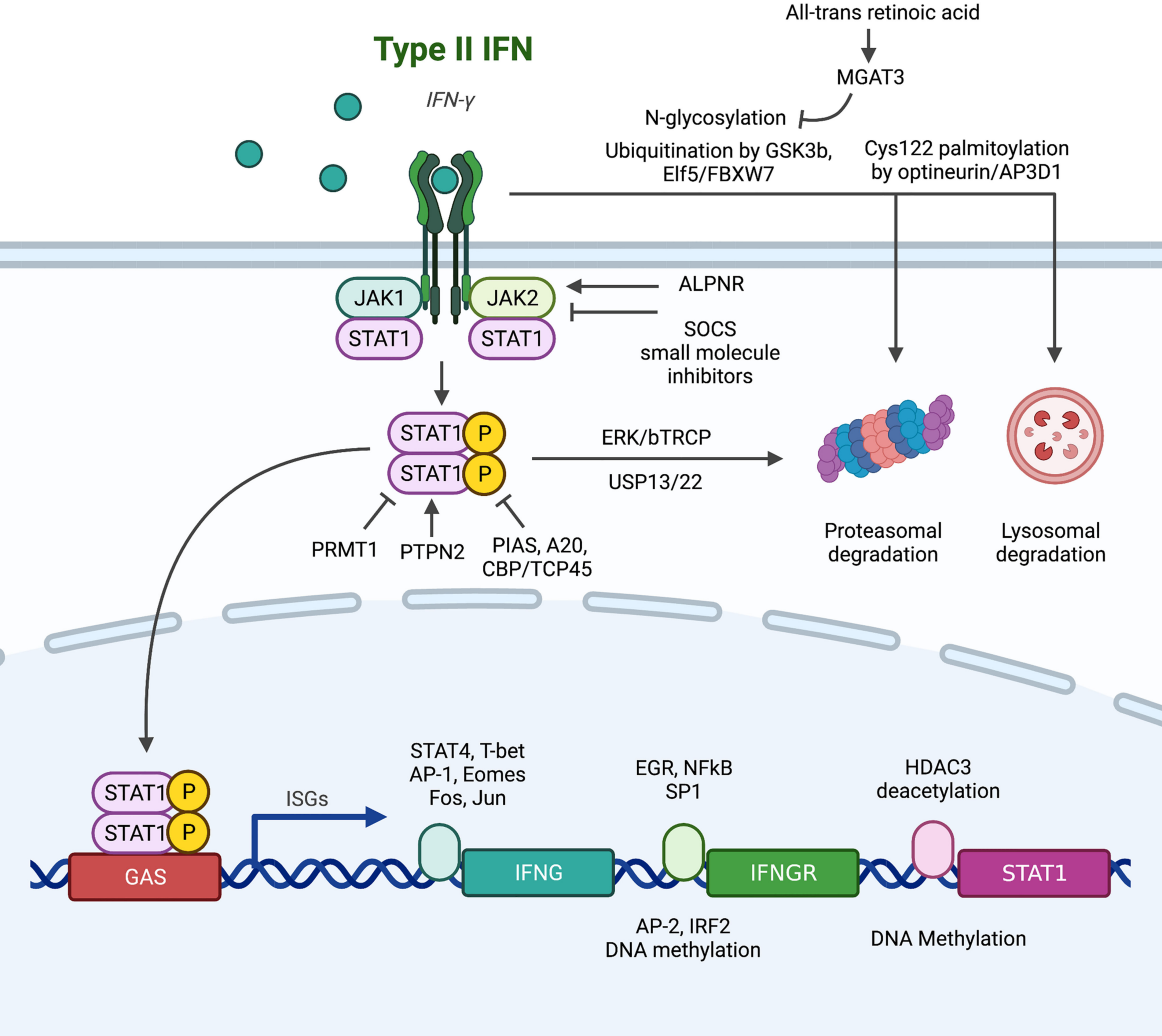
Summary of alterations in the conventional downstream signaling pathway of IFNGR/JAK/STAT that lead to IFN-γ resistance. (1) At the IFNGR level: transcriptionally, EGR, NFκB and SP1 promote the transcription of IFNGR mRNA, while AP2 and IRF2 are suppressive transcription factors. Post-transcriptionally, N-glycosylation stabilizes plasma membrane IFNGR, while degradation is promoted by ubiquitination and palmitoylation. (2) At the JAK level, SOCS and small molecule inhibitors abrogate signaling, and ALPNR is crucial for normal functioning. (3) At the STAT1 level, post-translational modification by PIAS, A20, and CBP/TCP45 suppress signaling activity through deposphorylation of p-STAT1, while PRMT1, ERK/bTRCP, and USP13/22 regulate STAT1 activity or stability independent of its phosphorylation status.

The canonical pathway of IFNGR/JAK1/STAT1/ISGs is the main effector downstream of IFN-γ signaling. Signal transduction along this pathway is affected by genomic mutation, transcriptional regulation of expression, post-translational modifications and protein-protein interaction, and sub-cellular localization (**Figure 1**), along with further complexity due to homologous members of the same family with overlapping but distinct functions (7, 61–63).

### IFNGR

IFN-γR is composed of two subunits: IFNGR1 (α-subunit) has high affinity to IFN-γ and a major role in ligand binding, while IFNGR2 (β-subunit) is predominantly responsible for downstream signaling *via* JAK recruitment (61, 64). Upregulation of IFNGR on colorectal cancer stem cell confers sensitivity to chemotherapy through apoptosis (56), but increased IFNGR expression on CD8+ T cells leads to T cell apoptosis and ICB resistance (38). Th1 cells can downregulate IFNGR to enhance survival and maintain antitumor effects (28), but tumor cells also developed multiple mechanisms to abrogate IFNGR function. At the **genomic level**, IFNGR has high mutation frequency of 12%, comparable with JAK1, JAK2, and IRF1, in melanoma patients resistant to anti-CTLA4 therapy (65). At the **transcription level**, IFN-γR is under dynamic regulation by transcription factors. EGR and NFkB (66) and SP1 in breast cancer (67) have been reported to induce the mRNA expression of IFNGR, while AP2 in breast cancer (67) and IRF2-mediated negative feedback in esophageal cancer (68) have suppressive roles. Chronic IFN-γ stimulation in colorectal cancer cell line mounts IFNGR resistance through DNA methylation, a process fully reversible by 5-Aza-deoxycytidine (69). At the **post-translational level**, IFNGR dynamically localizes between plasma, endosomal and nuclear membranes, leading to three potential fates: 1) recycling back to plasma membrane for further signaling, 2) degradation to attenuates function, 3) nuclear translocation. The degradation of IFNGR is under extensive regulation. IFNGR is subjected to degradation by the proteasome through GSK3b (70), ELF5/FBXW7 (46) and N-glycosylation (reversible by all-trans retinoic acid induces MGAT expression) (71) mediated ubiquitination in monocytic cell lines, breast cancer, and colorectal cancer respectively. Optineurin/AP3D1 mediated Cys122 palmitoylation targets IFNGR for lysosomal degradation in colorectal cancer (72). Furthermore, the nuclear import of IFNGR has been recently reported be functionally significant in breast cancer (51), mechanistically by bringing STAT1 into the nucleus (73–75) and direct GAS binding (76, 77).

### JAKs

As IFNGR lacks intrinsic kinase activity and only functions as a scaffold upon dimerization induced by IFN-γ, the four members of tyrosine kinase adaptors, JAK1, JAK2, JAK3, and TYK2, are needed to carry out subsequent signaling by recruitment and phosphorylation of STAT1. **Loss-of-function mutations** in JAK1 and JAK2 are extensively reported to be the cause of ICB resistance in melanoma patients (65, 78–82) and gynecologic cancers (83). JAKs are also the primary brake for **negative regulation** of IFN-γ activity by protein-protein interactions. The well-studied suppressor of cytokine signaling (SOCS) proteins bind to activated JAK catalytic sites (84) and are constantly active in melanoma cell lines to suppress interferon response (85). Disruption of APLNR-JAK1 interaction (81) both capable of abrogating IFN-γ activity. However, JAKs are often reported to have pro-tumor roles, highly expressed and correlated with poor survival in pancreatic cancer (86). The activating mutation (S703I) of JAK1 elevates p-STAT3 and STAT5 in liver cancer, driving tumor progression (87). Hence, lots of **JAK inhibitors** have been developed and put under clinical testing (88, 89). A phase II study (NCT01423604) of ruxolitinib and capecitabine/gemcitabine improved survival of metastatic pancreatic cancer patients with CRP>13 mg/L (90), and a phase Ib/II study (NCT01858883) of itacitinib and nab-paclitaxel and gemcitabine achieved objective response rate of 24% in patients with solid tumors (91). However, subsequent Phase III trials JANUS 1 (NCT02117479) and JANUS 2 (NCT02119663) (92), along with phase I trials of other agents including the selective JAK1 inhibitor INCB047986 (NCT01929941) (89) and momelotinib + gemcitabine and nab-paclitaxel (NCT02101021) (93) or capecitabine and oxaliplatin (NCT02244489), have all been terminated due to lack of efficacy. Despite much failure, there are still ongoing studies awaiting results, such as the phase Ib study of ruxolitinib and trametinib (MEK inhibitor) in patients with RAS mutations (NCT04303403). Another agent, AG490, was effective in preclinical model of mouse pancreatic cancer but awaits clinical validation (94). The challenge in clinical application of JAK inhibitors suggested the need for more specific interventions such as STAT3 inhibitors (95, 96), or better selection of patients that may benefit from JAK inhibitors.

### STATs

The **seven members** of the STAT family, STAT1, STAT2, STAT3, STAT4, STAT5A, STAT5B and STAT6, are often simultaneously activated, leading to diverse, cell-specific effects depending on abundance and availability of each member. Among them, STAT3 and STAT5 are often considered pro-tumor (97), while **STAT1** is generally considered the key downstream effector of IFN-γ, capable binding GAS and inducing ISG transcriptions. STAT1 has been reported to be protective in gastric cancer (98), liver cancer (99), and melanoma (100), but hazardous in glioblastoma (101) and sarcoma (102), and controversial in breast (103–106) and pancreatic cancer. STAT1 is under extensive **post-translational modifications**: 1) STAT1 is targeted for proteasomal degradation by ERK/bTRCP (107, 108) and stabilized by USP13 (109) and USP22 (110) mediated deubiquitination; 2) aberrant phosphorylation of STAT1 due to negative regulators PIAS (111), A20 (112) and CBP/TCP45 (113); and 3) functional impairment due to arginine methylation by PRMT1 (114) and loss of the helper PTPN2 (115). Interestingly, unphosphorylated STAT1 (u-STAT1) is also functional (116). Marked by persistence for several days post-IFN-γ stimulation, u-STAT1 are reservoir for phosphorylation-activation, allowing IFN-γ stimulation to prime for IFN-α response (117, 118). u-STAT1 is also responsible for the constitutive baseline expression caspases, conferring sensitivity to apoptotic signals (119). These functions of u-STAT1 further suggest the importance of **expression control** by epigenetic modifications of the STAT1 promoter: suppressed by methylation (120) and enhanced by HDAC3 mediated deacetylation (121).

## ISGs

**Figure 2:**
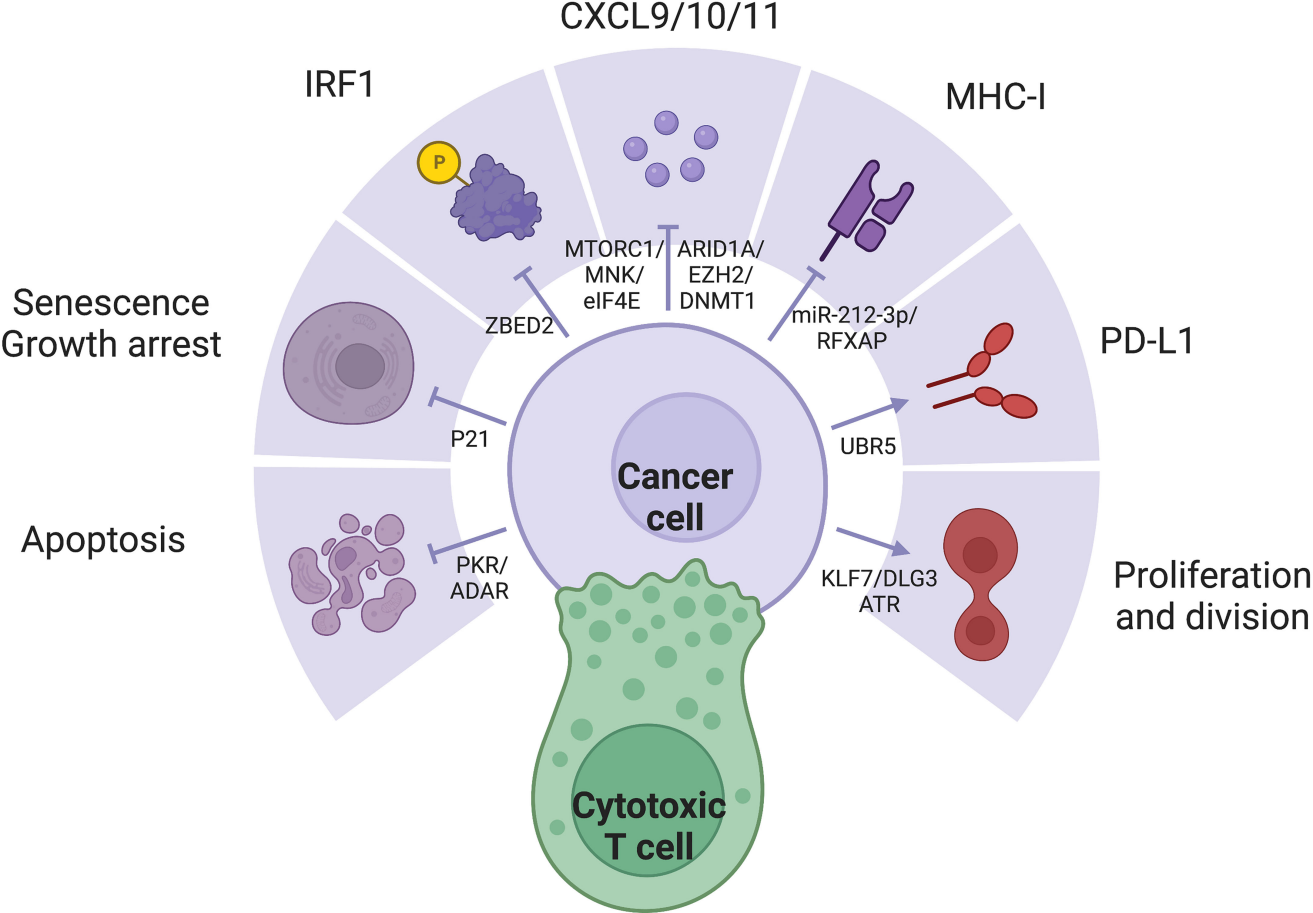
IFN-γ exerts a variety of effects through diverse expression pattern of ISGs, which is often dysregulated in cancer cells: (1) IRF1 is a major downstream effector of IFN-γ with diverse functions. (2) IFN-y represses tumor through apoptosis, senescence, growth arrest, secretion of inflammatory cytokines, and MHC-I expression. (3) IFN-γ signaling may also promote tumor through proliferation and PD-L1 expression.

IFN-γ stimulation changes the expression of hundred of genes, collectively producing a wide spectrum of effects. The large number and dynamic nature of ISGs render a consensus definition challenging. It is important to acknowledge the context-specific expression pattern of ISGs, who spearhead changes in cellular pathways and activities, directly corresponding to the effector functions of IFN-γ (**Figure 2**). Several key members of ISGs with important functional consequences, such as IRF1, CXCL9/10, MHC-I/II, and PDL1, have been extensively studied. The general pattern and mechanism behind the simultaneous expression of multiple ISGs must also be kept in mind, as simple protein-protein interactions may be insufficient to characterize such extensive regulation. Epigenetic remodeling such as histone and promoter modifications, chromatin conformation and accessibility, transcription-factor binding, latent enhancer and promoter regulations have crucial but rather unelucidated roles (122).

### Definition

Being able to clearly define ISGs would aid in selecting the appropriate therapeutic target. There are currently two approaches: in silicon or *in vitro*. The in silicon definition of ISGs rely on GAS, a consensus, palindromic DNA pattern (TTCN2-4 GAA) found in the promoters of ISGs. Its recognition and binding by p-STAT1 dimer produce profound effect in modulating the expression of its downstream genes. Hence, the SABioscience website, the Transcription Factor binding site search tools and the REFINEMENT program can identify GAS distributed throughout the human and mouse genome, theoretically denoting the downstream genes as ISGs (123). *In vitro* definitions of ISGs are primarily based on high-throughput characterization of gene expression changes after IFN-γ stimulation. Microarray analysis identified more than 300 ISGs assigned into categories (124). Human fibrosarcoma cell line treated with IFN-α, IFN-β and IFN-γ identified shared and distinct ISGs between these classes (2). ISGs have also been characterized in the Huh7 and Huh7.5 hepatocellular cell lines (125). However, these results are heavily dependent on the model and background, limiting their generalizability. The large number of ISGs, dynamic and context-dependent expression profile affected by epigenetic modification and chromatin remodeling, the degeneration and non-redundancy between the three classes of IFNs (126), and different STAT and IRF isoforms all pose challenges to a consensus definition of ISGs (127).

### Key members

Interferon regulatory factors (IRFs) are major ISGs (128). The nine members of the IRF family share similar structures. The homologous N-terminal DNA-binding domain (DBD) recognizes the helix-loop-helix motif of IRF-element (IRFE) motifs upstream of their effector genes; while the C-terminal, responsible for protein interaction confer the diversity of regulation. IRF1/5/6/8/9 have been reported to be protective and pro-apoptotic, while IRF2/3/4/7 are often considered hazardous (20). Among the IRF members, **IRF1** is the major driver of the expression of many ISGs (129, 130). With a GAS but not ISRE in its promoter, IRF1 is induced by IFN-γ rather than IFN-α and IFN-β, as a major distinction between the effects of type I and II IFNs (131). IRF8, whose expression is often jointed with IRF1, enhances the pro-apoptotic effect of IRF1 (132). IFN-γ exerts **antiproliferative** effects through cell cycle control and cell death induction. IFN-γ mainly depends on STAT1 activity to induce pro-death molecules including p53 in ovarian cancer (133, 134), caspase 1/3/8 in pancreatic cancer (135–137) and FAS/FASL (138), and the necroptotic RIP1 (47). IFN-γ also arrest growth through p16/p21-induced senescence (48, 51, 139) and inhibition of PI3K/AKT pathway (140). IFN-γ increases **antigenicity** through antigen processing and presentation, upregulating both MHC-I/II themselves (141, 142), as well as antigen processing machinery including TAP1/2, invariant chain, and the expression and activity of the proteasome (143), and the MHC class II transactivator (CIITA) (144). IFN-γ pathway directly transcribes pro-inflammatory **cytokines** especially CXCL9/10/11 (145). However, IFN-γ also induces the expression of the ICBs including but not limited to PDL1 and CTLA4 (146, 147), along with other **immunosuppressive** mechanisms such as IDO (148). Favorable expression of these key ISGs with drastically different functions profoundly influence the outcome of IFN-γ stimulation.

### Regulation

The importance of the SWI/SNF complex mediated chromatin remodeling in IFN-y response was further supported by its crucial rolel in the coordination of T cell activation and exhaustion (149). RNF138 mediated K48-linked polyubiquitination at position Lys643 of SMARCC1 drives proteasomal degradation, inhibiting chromatin remodeling at SWI/SNF-regulated gene loci, suppress transcription of late inflammatory genes of macrophages in innate immune activation (150). On a global scale, differences in genomic accessibility due to **chromatin remodeling** allow the preferential expression of some ISGs over others. In melanoma cell lines, the PBAF form of the SWI/SNF chromatin remodeling complex suppresses IFN-γ signaling and cytokine gene transcription to resist T cell infiltration and cytotoxicity (151). Employment of CRISPR screening, Chip-seq and ATAC-seq may further identify other regulatory mechanisms of chromatin remodeling in different models (126), shedding light on comprehensive. More **specifically**, many molecules have been reported to selectively affect key ISGs, rendering them potential therapeutic targets for precise modulation of IFN-γ response. ZBED2 antagonizes IRF1 and drives T cell dysfunction (4, 152). Important antitumor cytokines CXCL9 and CXCL10 are negatively regulated by epigenetic silencing by EZH2/DNMT1 in ovarian cancer and antagonized by ARID1A (153, 154), but activated by mTORC1/MNK/eIF4E (32). Inability to upregulate MHC I expression has been the earliest reported sign to IFN resistance reported in 33% melanoma cell lines and 24% lung adenocarcinoma cell lines (155). MHC-II is suppressed by c-myc activity (156), miR-212-3p through inhibition of RFXAP (157) and DNMT1/3B mediated DNA methylation that epigenetically inactivates CIITA (158–160), as a crucial limiting factor to tumor antigenicity (161). UBR5 promotes IFN-γ and STAT1 dependent PDL1 expression (162, 163), leading to combination of IFN-γ and nivolumab achieved the best response (164). KLF7/DLG3 maintains Golgi integrity and expression of pro-tumor ISGs (165). U-STAT1 expression without Y701 phosphorylation induce MUC4 expression in PDAC cells and exerts pro-EMT functions (166). ATR mediated nucleotide metabolism help maintain survival despite IFN stimulation (167). The dsRNA-activated kinase PKR confers lethality by IFN stimulation, a process antagonized by the dsRNA editing enzyme ADAR, which maintains survival despite the expression of death-inducing ISGs (168). IFN-γ activates the RhoGDI2/Rac1/NF-κB pathway in tumor cells to reduce the production of the pro-tumor cytokine CXCL8, promoting tumor apoptosis (169) and reducing CXCR2+ M2 macrophages (170). Targeting of tumor cell–intrinsic resistance mechanisms to T cell-mediated cytotoxicity is important and complementary to checkpoint inhibitors.

## Therapeutics

### Prognostic

IFN-γ activity is necessary and prognostic in immunotherapy of multiple cancer types (171). At the genomic level, mutation of key molecules along the IFN-γ signaling pathway are common in ICB refractory patients with melanoma (65), lung cancer (172) and gastric cancer (173). At the transcriptomic level, high expression of IFN-γ itself and IFN-γ responsive genes, reflective of a T cell-inflamed microenvironment, correspond to better survival and ICB response across different cancer types (174), especially lung cancer (175) and melanoma (176, 177). Aberrant attenuation of the IFN-γ pathway confers ICB resistance through several mechanisms, including both primary resistance by suppression of PDL1 expression (79) and acquired resistance by MHC-I downregulation (78).

### Cellular therapy

IFN-γ is especially important in cellular immunity and adoptive cell transfer. Patients with pancreatic cancer have decreased plasma IFN-γ compared to normal people (averaged 16.4 fg/L versus 27.4 fg/L), potentially leading to low CD4:CD8 ratio and systemic immunosuppression (178). Higher plasma IFN-γ after TILs administration is associated with prolonged T cell persistence and better survival (179). The cytotoxicity of T and NK cells heavily depends on IFN-γ secretion (180–182). In the context of CAR-T cells, IFN-γ secretion upon activation (183) not only exerts self-sustaining effects but also establishes cytokine crosstalk with endogenous T and NK cells (26, 184). Interestingly, tumor response to IFN-γ, such as APLNR interaction with JAK1 in melanoma cells (81), is crucial for CAR-T cytotoxicity only in solid tumors but not hematological tumors (185). IFN-γ abrogation offers protection against CRS (186) without impairing the therapeutic efficacy of anti-CD19 CAR-T in leukemias or lymphomas (187). CAR-T with an excessively high IFN-γ expression has been reported to upregulate PD-L1 expression in cancer cells, leading to their own dysfunction (188).

### As therapeutic agent

The clinical application of IFN-γ covered a wide variety of diseases such as cancer, infectious diseases, and autoimmune disorders (189). The antitumor efficacy of direct IFN-γ administration has long been debated. Initial attempts in renal cell carcinoma was disappointing (190), but later studies in prostate cancer (191) and bladder cancer had promising results (73.4% versus 57.2% with no recurrence during follow-up) (192). IFN-γ in ovarian cancer had controversial results: 2/6 patients experienced a 90% reduction in tumor cells in ascites (193), but limited responses have also been reported (194, 195).

The short half-life and cost of protein production led to much effort into the optimization of IFN-γ products, aiming to minimize adverse effect, amplify antitumor activity, and optimize cost and convenience of delivery. Apart from clinically available forms including the recombinant protein (IFN-γ1b, Actimmune), adenovirus vectors that express IFN-γ cDNA (TG-1041, TG-1042), and neutralizing antibodies against IFN-γ (HuZaf and AMG811), researchers have also reported: 1) engineered chimeric proteins such as chTNT3/muIFN-y (196), PLGF2/IFN-α (197), PDGFbR/IFN-γ (198) aiming to achieve specific and targeted delivery; 2) liposome- and nonmaterial-aided delivery (199) to reduce DNA damage and oxidative stress in lymphocytes; 3) as adjuvant treatment in conjugation with oncolytic viruses (200) and adoptive cell therapy; 4) loaded into cellular therapy, such as IFN-γ-secreting stromal cells with TME-homing capacities (201), and fourth generation CAR-T cells (TRUNKS) engineered to secrete IFN-γ constitutively or upon activation.

## Perspectives

IFN-γ is the central cytokine in anti-tumor immunity, not only indispensable for its supportive role in immune cell maturation and activation, but more importantly as an effector function. Increased antigenicity and apoptosis due to IFN-γ stimulation are the foundation of T cell cytotoxicity. However, tumor cells can take advantage the extensive regulatory mechanisms along the IFN-γ signaling pathway and the diversity of ISGs, favoring the expression of pro-tumor ISGs over others. Elucidating the mechanisms by which tumor cells hijack the IFN-γ pathway to suppress antitumor response including MHC-I and CXCL9/10/11expression, while preferentially enhancing the expression of immunosuppressive molecules such as PD-L1 and IDO1, would shed light on the conflicting identify of IFN-γ in tumor progression, along with providing therapeutic targets to improve current immunotherapies. In summary, tumor cells can mount IFN-γ resistance at two stages: disruption of the main signal transduction pathway responsible for all downstream effects of IFN-γ stimulation, and the alternative expression/suppression of specific key ISG or a group of ISGs with synergistic functions. Tumors cells can abrogate the IFNGR/JAK/STAT pathway through the following mechanisms: 1) loss of function mutation of key genes, most commonly IFNGR1/IFNGR2/JAK1/JAK2; 2) suppressed mRNA transcription of IFNG, IFNGR, and STAT1 leading to lower expression; 3) loss of crucial protein cooperators such as ALPNR/JAK1, PTPN2/STAT1; 3) aberrant hyperactivity of protein inhibitors such as SOCS, PIAS, and PRMT1; 4) post-translational modification, especially ubiquitination, subjecting the protein to degradation by the proteasome or lysosome. Further downstream, tumor cells can preferentially express some ISGs over others through 1) global chromatin remodeling and 2) specific modulations of key ISGs.

IFN-γ based therapies face several challenges that limit effectiveness: the development of resistance by tumor cells, and general adverse effects due to non-specific, systemic delivery. These mechanisms of IFN-γ resistance described above offer promising therapeutic targets to fine-tune IFN-γ response and improve immunotherapy. However, as these results were mostly based on experimental models, further confirmation and validations in real-world clinical data would provide valuable insights on the prevalence and significance of these potential targets.

## Author contributions

JH and MW contributed equally to this work. All authors contributed to the article and approved the submitted version.
